# Effect of Clinical Complete Remission Following Neoadjuvant Pembrolizumab or Chemotherapy in Bladder-Preservation Strategy in Patients with Muscle-Invasive Bladder Cancer Declining Definitive Local Therapy

**DOI:** 10.3390/cancers16050894

**Published:** 2024-02-23

**Authors:** Pei-Hung Chang, Hung-Yi Chen, Yueh-Shih Chang, Po-Jung Su, Wen-Kuan Huang, Cheng-Feng Lin, Jason Chia-Hsun Hsieh, Chun-Te Wu

**Affiliations:** 1 Division of Hematology Oncology, Department of Internal Medicine, Keelung Chang Gung Memorial Hospital, Keelung 20401, Taiwan; ph555chang@gmail.com (P.-H.C.);; 2 School of Medicine, College of Medicine, Chang Gung University, Taoyuan 333323, Taiwan; medfox0924@cgmh.org.tw; 3 Division of Urology, Department of Surgery, Keelung Chang Gung Memorial Hospital, Keelung 20401, Taiwan; hongyi@cgmh.org.tw (H.-Y.C.);; 4 Institute of Clinical Medicine, National Yang Ming Chiao Tung University, Taipei 112304, Taiwan; 5 Division of Hematology Oncology, Department of Internal Medicine, Linkou Chang Gung Memorial Hospital, Taoyuan 333423, Taiwan; pjsu@cgmh.org.tw (P.-J.S.); wisdom5000@gmail.com (J.C.-H.H.); 6 Division of Hematology Oncology, Department of Internal Medicine, New Taipei Municipal TuCheng Hospital, New Taipei 236017, Taiwan; 7 Division of Urology, Department of Surgery, Linkou Chang Gung Memorial Hospital, Taoyuan 333423, Taiwan

**Keywords:** bladder preservation, neoadjuvant chemotherapy, pembrolizumab, muscle-invasive bladder cancer, clinical complete remission

## Abstract

**Simple Summary:**

This study focused on patients with muscle-invasive bladder cancer initially planned for neoadjuvant systemic therapy followed by cystectomy, but who later declined cystectomy or other definitive local therapy, opting for conservative management. This retrospective analysis aimed to assess disease and bladder-preservation outcomes in this specific cohort. The therapeutic approach involved maximal transurethral resection of the bladder tumor followed by either neoadjuvant chemotherapy or pembrolizumab. The primary objective was to evaluate the efficacy of these treatments and identify potential predictors for optimal patient outcomes. The preliminary findings suggest that pembrolizumab may offer superior outcomes compared to standard chemotherapy. Notably, patients achieving clinical complete remission after neoadjuvant treatment demonstrated significantly improved survival rates. These results may influence future therapeutic recommendations, advocating for more tailored and conservative approaches to managing muscle-invasive bladder cancer in patients unwilling to undergo cystectomy or other definitive local therapies. Bladder preservation, particularly following neoadjuvant complete remission, appears effective, and pembrolizumab emerges as a promising option for those unsuitable for chemotherapy.

**Abstract:**

This study aimed to evaluate the outcomes and identify the predictive factors of a bladder-preservation approach incorporating maximal transurethral resection of bladder tumor (TURBT) coupled with either pembrolizumab or chemotherapy for patients diagnosed with muscle-invasive bladder cancer (MIBC) who opted against definitive local therapy. We conducted a retrospective analysis on 53 MIBC (cT2-T3N0M0) patients who initially planned for neoadjuvant pembrolizumab or chemotherapy after maximal TURBT but later declined radical cystectomy and radiotherapy. Post-therapy clinical restaging and conservative bladder-preservation measures were employed. Clinical complete remission was defined as negative findings on cystoscopy with biopsy confirming the absence of malignancy if performed, negative urine cytology, and unremarkable cross-sectional imaging (either CT scan or MRI) following neoadjuvant therapy. Twenty-three patients received pembrolizumab, while thirty received chemotherapy. Our findings revealed that twenty-three (43.4%) patients achieved clinical complete response after neoadjuvant therapy. The complete remission rate was marginally higher in pembrolizumab group in comparison to chemotherapy group (52.1% vs. 36.7%, *p* = 0.26). After a median follow-up of 37.6 months, patients in the pembrolizumab group demonstrated a longer PFS (median, not reached vs. 20.2 months, *p* = 0.078) and OS (median, not reached vs. 26.8 months, *p* = 0.027) relative to those in chemotherapy group. Those achieving clinical complete remission post-neoadjuvant therapy also exhibited prolonged PFS (median, not reached vs. 10.2 months, *p* < 0.001) and OS (median, not reached vs. 24.4 months, *p* = 0.004). In the multivariate analysis, clinical complete remission subsequent to neoadjuvant therapy was independently associated with superior PFS and OS. In conclusion, bladder preservation emerges as a viable therapeutic strategy for a carefully selected cohort of MIBC patients without definitive local therapy, especially those achieving clinical complete remission following neoadjuvant treatment. For patients unfit for chemotherapy, pembrolizumab offers a promising alternative treatment option.

## 1. Introduction

The current gold standard treatment for muscle-invasive bladder cancer remains radical cystectomy (RC) with neoadjuvant chemotherapy (NAC) [1]. While RC is effective, it carries a wide range of potential complications that can markedly impair a patient’s quality of life, owing to alterations in urinary and, at times, sexual functionalities. Furthermore, it comes with a high morbidity rate (66%) and a significant risk of mortality during the perioperative period (4.2%) [2,3]. Considering the significant risk of perioperative hazards and the consequential decrements in life quality related to RC, the investigation of bladder-preservation strategies is imperative.

Cisplatin-based NAC followed by RC significantly improves overall survival as evidenced by numerous randomized controlled trials and meta-analyses and is recommended by contemporary international guidelines [4,5,6]. Several studies have indicated that the achievement of a pathological complete response (pCR) after NAC, meaning that there is no residual tumor at the primary site and within pelvic lymph nodes in the cystectomy specimen, correlates with positive long-term survival outcomes. The 5-year OS rate approximates to 80% for patients manifesting pCR [4,7,8]. Among those achieving pCR post-NAC, up to 40% might be suitable candidates for bladder preservation [7]. Yet, the only way to definitively verify pCR status after NAC is through a radical cystectomy [5,7]. Therefore, the noteworthy 30–40% rate of pathological complete response to NAC prompts the consideration of whether these patients could benefit from rigorous clinical monitoring instead of facing the potential complications associated with RC. Clinical complete response (cCR) following neoadjuvant chemotherapy (NAC) is characterized by the absence of residual tumors upon cystoscopy, transurethral resection (TUR), urine cytology, and cross-sectional imaging [5]. Should the prognostic significance of cCR be robustly validated as a promising alternative endpoint, the precise determination of cCR for NAC before radical cystectomy (RC) has the potential to instigate a transformative shift in bladder-preserving approaches for managing muscle-invasive bladder cancer.

Recent advances in immune checkpoint inhibitors (ICIs) have introduced a new era in bladder cancer treatment. Their potential as neoadjuvant treatments is also being investigated [9]. In the PURE-01 study, 42% of participants who received pembrolizumab as neoadjuvant therapy reached pCR [10]. Consequently, pembrolizumab may present a viable therapeutic alternative for patients who are unfit for chemotherapy in the neoadjuvant setting. Several researchers have reported the outcomes of neoadjuvant chemotherapy, demonstrating high survival rates with bladder preservation [11,12,13,14]. However, there are few studies on bladder preservation after neoadjuvant immunotherapy. In the present study, we retrospectively evaluated the clinical outcomes of MIBC patients who were initially planned for neoadjuvant systemic chemotherapy or pembrolizumab therapy followed by cystectomy but later declined definitive local therapy, including RC and radiotherapy, opting for conservative management. Additionally, we endeavored to delineate specific patient subgroups optimally positioned for bladder preservation via rigorous active surveillance.

## 2. Materials and Methods

### 2.1. Participants

We conducted a retrospective investigation on patients diagnosed with clinical T2-3N0M0 muscle-invasive bladder urothelial carcinoma at the Chang Gung Medical Foundation of Keelung Chang Gung Memorial Hospital, Taiwan. These patients had initially undergone maximal TURBT and were treated with either chemotherapy or pembrolizumab as neoadjuvant therapy in preparation for a planned radical cystectomy. However, they opted against undergoing radical cystectomy post-neoadjuvant therapy at our facility. Maximal transurethral resection of the bladder tumor (TURBT) and/or complete tumor resection is our routine practice, regardless of whether patients may undergo cystectomy later. Definitive radiotherapy or chemoradiotherapy were routinely offered at our facility for those who chose not to undergo radical cystectomy. However, this retrospective analysis aimed to assess disease and bladder-preservation outcomes without definitive local therapy in this specific cohort; therefore, we excluded patients who had undergone definitive radiotherapy or chemoradiotherapy. The patient recruitment spanned from January 2015 to December 2022.

For all patients, the collected data encompassed patient age, sex, TNM stage, Eastern Cooperative Oncology Group (ECOG) performance status, pathology, programmed death ligand-1 (PD-L1) status, overall survival (OS), and progression-free survival (PFS). Tumor staging adhered to the American Joint Committee on Cancer Staging System 7th edition (AJCC 7th edition), drawing from TURBT pathology results, imaging (CT or MRI), physical evaluations, chest radiographs, and standard routine laboratory tests. Complete and maximal TURBT was performed for all patients to resect all visible disease, ensuring the inclusion of muscularis propria, which was histopathologically confirmed before the administration of neoadjuvant therapy. Pathological diagnoses of all patients were evaluated by our institutional urological malignancy committee, which comprises three medical oncologists, three urologists, two pathologists, and two radiologists. Upon MIBC diagnosis confirmation, patients were directed for neoadjuvant therapy. The precise regimen was determined by the attending medical oncologist. In principle, patients were initially offered neoadjuvant chemotherapy as the primary treatment modality, with options such as gemcitabine plus cisplatin or carboplatin. For patients deemed unfit for chemotherapy or those who declined it, pembrolizumab was recommended as an alternative. The initial decision regarding the choice of regimen did not hinge on PD-L1 status. The specific regimens consist of gemcitabine (1000 mg/m^2^ on days 1, 8) plus cisplatin (70 mg/m^2^ on day 1) given every 21 days for 4 cycles, gemcitabine (1000 mg/m^2^ on days 1, 8) plus carboplatin (area under the curve 5) for 4 cycles, or pembrolizumab (200 mg) every 21 days for 3 cycles. Those who were cisplatin ineligible were defined by (1) ECOG performance status of 2 and/or (2) creatinine clearance < 60 mL/min and/or (3) CTCAE Gr ≥ 2 hearing loss and/or (4) CTCAE Gr ≥ 2 neuropathy. Maintenance pembrolizumab was offered to patients who achieved clinical complete remission and refused further radical cystectomy. The planned total treatment with pembrolizumab was 17 doses (approximately 1 year), encompassing both the neoadjuvant and maintenance phases. Chemotherapy would be administered for a total of four doses in the neoadjuvant phase only. Treatment would continue until unacceptable toxicity or disease progression. Toxicities were graded by the Common Terminology Criteria for Adverse Events (CTCAE), version 5.0.

Treatment response was evaluated after the completion of neoadjuvant therapy. Clinical complete remission was defined as negative outcomes in urine cytology, cystoscopy, and cross-sectional imaging (CT scan or MRI) after neoadjuvant therapy. During visual endoscopic evaluation, if no residual tumor was observed, no biopsy was performed. However, if any suspicious residual or persistent tumor was identified, a biopsy was conducted. A negative cystoscopy finding was defined as the absence of malignancy by negative visual endoscopic evaluation and/or negative biopsy. Radiological complete remission, defined as the absence of any features of bladder cancer on post-neoadjuvant imaging, should be confirmed by two radiologists.

Patients who experienced non-invasive downstaging (defined as ypT1, ypTis, ypTa) underwent transurethral resection of bladder tumor (TURBT) followed by intravesical bacille Calmette–Guérin (BCG) therapy. Radiotherapy treatment options were offered to patients with persistent muscle-invasive bladder cancer; however, none of the patients received radiation therapy in this study. After confirming the status of clinical complete remission, patients were recommended that RC was the conventional standard therapeutic approach. None were pre-selected for bladder preservation; they declined RC after consultation. Patients who refused RC were subject to an intensive active-monitoring plan. This included cystoscopy, with optional biopsy, and urine cytology every 2–3 months. Additionally, they underwent CT scans every three months for the initial two years, every four months in the third year, and semi-annually for the following two years. After this period, yearly check-ups were scheduled for an indefinite duration. When faced with local recurrence, RC was reiterated as the primary therapeutic recommendation. If patients still declined RC, they were managed based on protocols for non–muscle-invasive bladder cancer (NMIBC), either TURBT alone or combined with bacillus Calmette–Guérin (BCG) based on their restaging assessment. Systemic therapies were prescribed for patients that recurred with metastatic disease.

We exclusively enrolled newly diagnosed cases of bladder urothelial carcinoma for analysis. Individuals with recurrent bladder cancer or those concurrently diagnosed with another active malignancy were excluded from consideration. Patients planned for definitive radiotherapy or chemoradiotherapy were also excluded from this study. Following these exclusions, the data from a cohort of 53 participants were assessed. This study was conducted in strict adherence to the ethical standards outlined by the Declaration of Helsinki and we secured approval from the institutional review board of Chang Gung Memorial Hospital (code: 202300872B0). 

### 2.2. Statistical Analysis

For categorical variables, we present patients’ demographic data as a number (%), and for continuous variables, we present them as median (range). Associations between clinical and pathological attributes across the two groups were assessed using the chi-square test. To evaluate age differences between the groups, we used the independent Student’s *t*-test (2-tailed). We defined OS as the time from the date of diagnosis to the date of death from any cause, and PFS as the time from the date of diagnosis to the first evidence of progression, encompassing local recurrence in both non-muscle-invasive bladder cancer (NMIBC) and muscle-invasive bladder cancer (MIBC), as well as distant progression. Patients were followed up until their death or until 30 May 2023. We conducted survival analysis using the Kaplan–Meier method and employed the log-rank test to assess differences. Additionally, we utilized the multivariate Cox’s proportional hazard model to examine the influence of independent factors on OS and PFS. The statistical analyses were executed using SPSS for Windows (version 18; SPSS Inc., Chicago, IL, USA) and were two-sided. Differences were deemed significant when a *p*-value of less than 0.05 was observed.

## 3. Results

Fifty-three patients were enrolled for analysis. The median age at diagnosis was 71 years (range 41–91), and 41 (77.4%) of the patients were male. The clinical stage before neoadjuvant therapy was T2 for 27 patients (50.9%) and T3 for 26 patients (49.1%). The majority of participants, 38 (71.7%), exhibited an Eastern Cooperative Oncology Group performance status between 0 and 1. Twenty-nine (54.7%) patients showed program death-ligand 1 (PD-L1) expression (defined as combined positive score (CPS) ≥ 10). Thirty (56.7%) patients were eligible for cisplatin treatment. Neoadjuvant therapy regimens were as follows: gemcitabine and cisplatin for 23 patients (43.4%), pembrolizumab for 23 patients (43.4%), and gemcitabine and carboplatin for 7 patients (13.2%). 

After neoadjuvant therapy, twenty-three patients (43.4%) achieved a clinical complete response. All 23 patients with complete clinical response (cCR) underwent thorough confirmation through repeat endoscopic examinations and biopsies, consistently confirming negative findings throughout the entire follow-up period of the study. Among the 30 patients with non-clinical complete responses, twenty-one patients (39.6%) achieved non-invasive down-staging (defined as ypT1, ypTis, and ypTa). Five patients (9.4%) remained stable, while four patients (7.5%) experienced disease progression. Out of the four patients experiencing disease progression, two developed distant metastases, while the remaining two exhibited progression to locally advanced muscle-invasive bladder cancer. Those who achieved non-invasive downstaging underwent TURBT followed by intravesical BCG therapy. Patients with stable or progressive disease after neoadjuvant therapy were offered salvage systemic therapy or radiotherapy, but none received further therapy. After a median follow-up of 37.6 months (range: 3–67 months), out of the 53 patients enrolled in the study, 25 (47.1%) experienced disease recurrence, 18 (34.0%) died, and 15 (28.3%) died of bladder cancer. The median time to recurrence and median time to death after neoadjuvant therapy were 20.1 months and 34.0 months, respectively. The median progression-free survival (PFS) was 24.5 months with a 95% confidence interval (CI) of 7.9–41.2 months, and the median overall survival (OS) was 37.6 months. We further analyzed the recurrence patterns in our cohort and found that 13 (52%) developed non-invasive bladder cancer (NMIBC) recurrence, 4 (16%) developed muscle-invasive bladder cancer (MIBC) recurrence, and 8 (32%) developed distant metastasis. Among the 23 patients who initially achieved clinical complete remission (cCR), 18 patients retained their bladder and remained without evidence of disease at the end of the study, with a median follow-up time of 27.0 months. Among these 18 patients, 11 were treated with pembrolizumab, while 7 underwent chemotherapy. Four patients developed non-muscle-invasive bladder cancer (NMIBC) recurrence, and one patient experienced distant metastasis.

We compared the clinical and tumor characteristics of the 53 patients, which included 23 pembrolizumab-treated patients and 30 chemotherapy-treated patients. These characteristics are listed in Table 1. No significant differences were observed in age, gender, stage, or ECOG performance status between the two groups. However, PD-L1 expression and cisplatin-ineligibility were significantly higher in the pembrolizumab group (Table 1). All the patients in the pembrolizumab group received at least three cycles of pembrolizumab, whereas 83.3% of patients completed four cycles of chemotherapy in the chemotherapy group. Treatment-related adverse effects (AE) are reported in Table 2. The most frequent AE in the pembrolizumab group was hypothyroidism, while thrombocytopenia was the most common in the chemotherapy group. No AEs higher than grade 3 (by CTCAE version 5.0) were observed in our cohort. Patients who achieved clinical complete remission and declined further radical cystectomy were offered maintenance pembrolizumab. The median number of maintenance cycles was 10 (range: 4–18).

**Figure 1:**
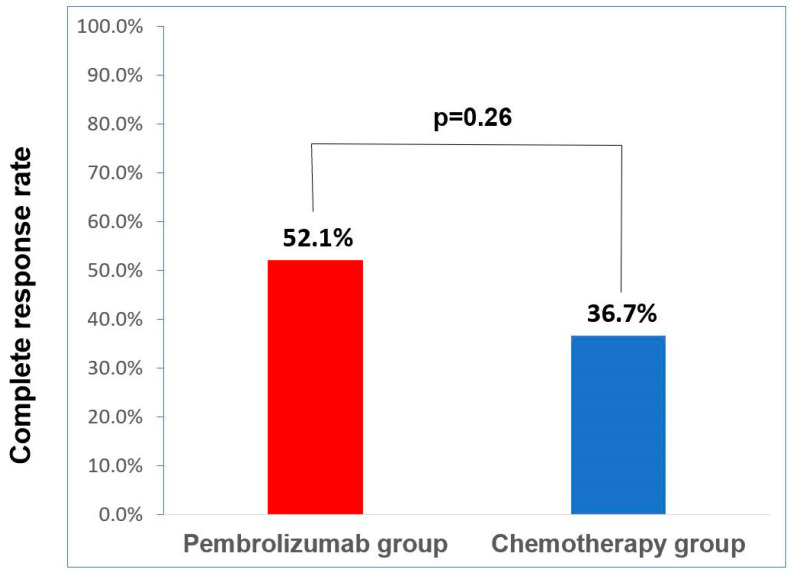
Clinical complete response rate stratified by pembrolizumab (*n* = 23) or chemotherapy (*n* = 30).

**Figure 2:**
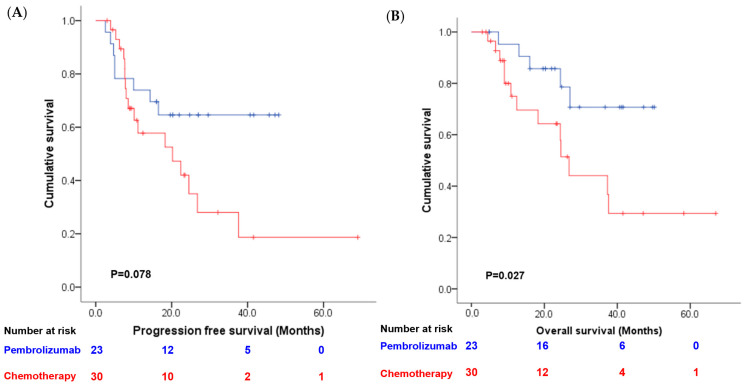
Comparison of (**A**) progression-free survival and (**B**) overall survival between patients treated with pembrolizumab (blue line) or chemotherapy (red line).

**Figure 3:**
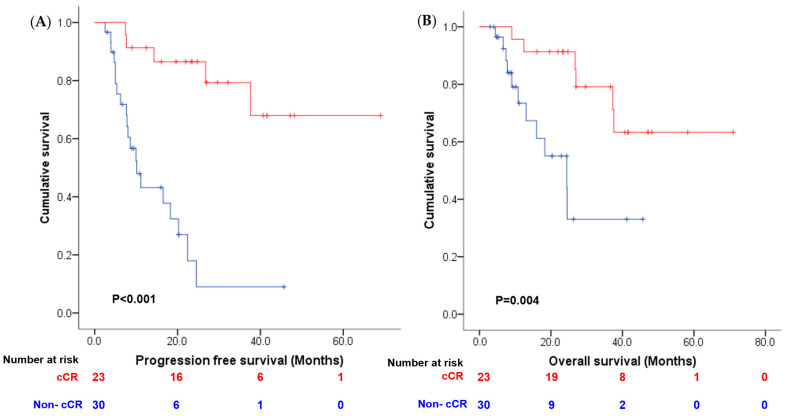
Comparison of (**A**) progression-free survival and (**B**) overall survival between clinical complete responder (red line) or non-complete responder (blue line).

As shown in Figure 1, the clinical complete remission rate was numerically higher in the pembrolizumab group than in the chemotherapy group (52.1%, 95% CI: 32.8–72.6% vs. 36.7%, 95% CI: 19.5–53.5%). However, this difference did not reach statistical significance (*p* = 0.26). We further analyzed the clinical complete remission rate according to PD-L1 status in the pembrolizumab group and found a trend toward a higher clinical complete remission rate in the high-PD-L1 group than in the low-PD-L1 group (61.1% vs. 20%, *p* = 0.1). We also compared the recurrence patterns between the chemotherapy and pembrolizumab groups and observed a higher rate of distant metastasis in the chemotherapy group (23.3% vs. 4.3%), although the difference was not statistically significant (*p* = 0.152) (Table 3). Patients in the pembrolizumab group showed a trend toward longer PFS (median not reached vs. 20.2 months, *p* = 0.078; see Figure 2A) and OS (median not reached vs. 26.8 months, *p* = 0.027; see Figure 2B) compared to patients in the chemotherapy group. Additionally, patients who achieved complete response after neoadjuvant therapy also had a longer PFS (median not reached vs. 10.2 months, *p* < 0.001; Figure 3A) and OS (median not reached vs. 24.4 months, *p* = 0.004; Figure 3B). Multivariate analysis revealed that achieving a clinical complete response after neoadjuvant therapy was an independent prognostic indicator for both PFS and OS when combined with other clinical parameters (Table 4). 

## 4. Discussion

Radical cystectomy is recognized for its effectiveness in treating MIBC. It achieves cancer control by removing the entire bladder and surrounding lymph nodes. However, due to its extensive nature, RC can lead to a range of complications—from immediate surgical risks to long-term lifestyle adjustments. Several studies have shown that pCR has a significant prognostic impact on survival [15]. However, pCR can only be confirmed after RC. As a result, cCR is then proposed as an additional measure endpoint for pCR to evaluate disease status before RC. This has paved the way for a new treatment approach, focusing on bladder-preservation strategies for patients who achieve cCR after neoadjuvant therapy [16]. Numerous studies suggest that MIBC patients who achieved cCR to NAC had exhibited high survival rates with bladder preservation. For instance, Herr reported a 5-year OS of 63% and bladder preservation in 54% of patients who reached cCR status post-NAC and opted against RC [11]. Sternberg et al. have observed that out of 104 patients, 49 achieved cCR after neoadjuvant chemotherapy, with the selected bladder-preservation cohort displaying a 5-year OS of 67% [12]. In another investigation, Robins et al. have determined that among 41 MIBC patients manifesting cCR post-NAC and declining RC, the 5-year cancer-specific survival stood at 87%, while the disease-free survival rate was 58% [13]. Furthermore, Mazza et al. [14] have reported the outcomes for 148 MIBC patients who, after demonstrating a complete clinical response to NAC, chose active surveillance. The 5-year OS, cancer-specific survival, and disease-free survival rates were 86%, 90%, and 64%, respectively. In contrast to prior investigations, our study incorporated patients treated with either NAC or pembrolizumab. The outcomes indicated a cCR rate of 43.4%. Moreover, among those achieving cCR, the 3-year OS and progression-free survival rates were 73.9% and 78.2%, respectively.

Participants in clinical trials often present with better overall health, potentially affecting the type and quality of chemotherapy administered. Cisplatin-based NAC appears to offer superior overall survival (OS) benefits for bladder cancer patients compared to carboplatin-based regimens in individuals contraindicated for cisplatin [17]. Notably, approximately half of the patients in clinical practices are unsuitable for cisplatin-based chemotherapy, attributable to multiple comorbidities, diminished renal function, and/or prior contraindications [18,19]. The possibility of employing checkpoint inhibitors in a neoadjuvant setting is promising, given their good tolerability and effectiveness, and has been widely investigated [20]. On the other hand, data from clinical trials may not accurately reflect real-world clinical scenarios. In our study, 43.3% were ineligible for cisplatin treatment and 28.3% had an ECOG performance status of two or higher at the time of diagnosis. Additionally, patients with a poor performance status (ECOG ≥ 2) were also considered unsuitable candidates for cystectomy. In such cases, an initial approach of systemic therapy, particularly with pembrolizumab, followed by a re-evaluation of the suitability for cystectomy, may represent a more appropriate treatment option. In our study, 69.6% of patients in the pembrolizumab group were cisplatin-ineligible, which is significantly higher than the chemotherapy group. Despite higher cisplatin-ineligible rates, patients in the pembrolizumab showed higher cCR rates and better survival outcomes compared with the chemotherapy group. This result suggests that neoadjuvant pembrolizumab could be a treatment alternative for patients unsuitable for chemotherapy.

Several recent studies have explored the potential role of incorporating immunotherapy into neoadjuvant strategies. A phase 2 study HCRN GU16-257 trial [21] included 76 patients with cT2-4aN0M0 cisplatin-eligible patients. All patients received the combination of anti-PD1 antibody (nivolumab) with chemotherapy (gemcitabine + cisplatin) for four cycles. Patients with a clinical CR were offered either a radical cystectomy or no cystectomy as per patient choice. The clinical CR rate was 43% at the median follow up of 30 months, and most patients elected to forgo cystectomy. Patients with a clinical CR had significantly better metastasis-free survival and overall survival, which is consistent with the current study. Another real-world, multicenter investigation by Hu et al. [22] suggests that neoadjuvant chemoimmunotherapy (tislelizumab + gemcitabine + cisplatin) outperforms neoadjuvant immunotherapy or chemotherapy in terms of achieving the highest response rate. Individuals who attain a pathological complete response following neoadjuvant treatments, coupled with maximal transurethral resection of the bladder tumor, may be considered suitable candidates for bladder preservation therapy. They reported that 33 patients undergoing bladder preservation therapy with maximal transurethral resection of the bladder tumor achieved disease-free survival (93.94%) after a median follow-up of 13 months. Both studies incorporated a combination of neoadjuvant immunotherapy and chemotherapy, specifically using gemcitabine and cisplatin. However, this regimen may not be suitable for cisplatin-ineligible patients. Remarkably, our study stands out as the first to investigate the role of single-agent immunotherapy, pembrolizumab, in patients with muscle-invasive bladder cancer who plan to undergo neoadjuvant therapy alone without local therapy for bladder preservation.

Several points merit further discussion in our study. First, PD-L1 expression was significantly higher in the pembrolizumab group. In the context of advanced/metastatic urothelial carcinoma, for example, Keynote-052 and Keynote-045 [23,24,25,26,27] elucidate the role of pembrolizumab in both first-line and subsequent treatment phases. Remarkably, both trials showed that patients exhibiting high PD-L1 expression responded better to pembrolizumab and had superior survival. Expanding our perspective to the neoadjuvant setting, the PURE-01 trial [28] further emphasized the significance of PD-L1 expression. This study demonstrated that PD-L1 expression stands out as the most potent predictor of sustained response following neoadjuvant immunotherapy followed by radical cystectomy. In our specific cohort, where PD-L1 high expression was significantly elevated in the pembrolizumab group, we discerned a noteworthy trend towards a higher rate of clinical complete remission (cCR) in the high PD-L1 group compared to the low PD-L1 group (61.1% vs. 20%, *p* = 0.1) within the subset of patients treated with pembrolizumab. This observation may underline the elevated cCR rate and favorable survival outcomes observed in the pembrolizumab-treated group compared to the chemotherapy cohort. PD-L1 status may further serve as a biomarker for clinicians in the selection of neoadjuvant regimens. Second, failure patterns differed between the chemotherapy and pembrolizumab groups, with the distant metastatic rate being lower in the pembrolizumab group (4.3% vs. 23.3%) compared to chemotherapy. Due to the strong correlation between distant metastasis and survival, these findings suggest that pembrolizumab may contribute to a reduced rate of distant failure, ultimately leading to improved survival. Although they did not reach statistical significance due to the small sample size, further studies with larger sample sizes should be conducted to confirm these important observations. Third, the observed disparity in favorable outcomes favoring the pembrolizumab arm may be attributed to differences in treatment strategies within our cohort. Patients in the pembrolizumab arm, specifically those with clinical complete remission (cCR), received maintenance pembrolizumab. In contrast, patients in the chemotherapy arm did not undergo any maintenance therapy. In the context of advanced and metastatic bladder cancer, avelumab, an anti-PD-1 antibody, has shown significant improvements in survival among patients with urothelial cancer, particularly in cases where the disease did not progress following first-line chemotherapy [29]. A notable advancement has been the recognition of the importance of programmed cell death receptor 1 (PD-1) and programmed cell death ligand 1 (PD-L1) expression in advanced bladder cancer. Data suggest that PD-L1 expression after neoadjuvant chemotherapy is associated with prolonged survival [30]. If this holds true, there arises an opportunity to design trials in which patients achieving cCR after chemotherapy subsequently undergo maintenance with immune checkpoint inhibitors for muscle-invasive bladder cancer. This approach, aimed at bladder preservation, becomes particularly relevant for those who decline radical cystectomy. To validate these findings, additional well-designed prospective studies are warranted. Additionally, James et al. [31] have demonstrated that maximal transurethral resection of the bladder tumor (TURBT) before neoadjuvant chemotherapy (NAC) is strongly associated with a complete pathological response at the time of radical cystectomy (RC). We performed maximal TURBT in the entire cohort before systemic therapy, a practice that may have enhanced our treatment efficacy. Finally, the Kaplan–Meier curve for PFS with pembrolizumab showed a sharp initial drop and subsequently crossed the chemotherapy curve before leveling off. This suggests that some patients did not respond well to pembrolizumab and experienced rapid progression. Caution should be exercised in adopting this in routine clinical practice before validation is performed in prospective clinical trials.

This research has certain limitations that warrant consideration. First, we only included a relatively small number of patients from one single medical institution, which might have restricted the impact of clinical variables on survival outcomes. The limited sample size might not fully capture the heterogeneity present in a larger, more diverse patient population, potentially limiting the external validity of our results. Furthermore, the restricted patient pool may have influenced the robustness of the statistical analyses, as smaller sample sizes can introduce variability and limit the ability to detect subtle but clinically significant effects. Second, our study was retrospective and non-randomized, which could impact the reliability of our conclusions. While our study design was suitable for generating hypotheses, it may not establish a causal relationship between variables. The findings are certainly limited by their retrospective nature; however, this limitation could inform the basis of trial designs examining immunotherapy-based bladder-preservation protocols. Additionally, pembrolizumab was not reimbursed by national health insurance in Taiwan. This financial barrier may have influenced patient selection, treatment decisions, maintenance or not, and follow-up care, potentially introducing a source of bias. As reimbursement policies and healthcare access can vary across regions, the external applicability of our results may be limited to settings with similar reimbursement scenarios. To address these limitations, we emphasize the necessity for additional large-scale, prospective research studies, aimed at minimizing the impact of confounding variables. This approach will provide a more reliable basis for drawing conclusions regarding the effectiveness and safety of pembrolizumab in our target population. Finally, cCR as a surrogate endpoint is also controversial, and discordance between cCR and pCR has been discussed [20]. The absence of uniform restaging protocols and a standardized definition of cCR restricts its broad clinical application and impedes ongoing research focused on multidisciplinary strategies for bladder preservation. Consequently, the clinical validity of cCR as a prognostic marker for long-term survival following neoadjuvant treatment in MIBC demands systemic, step-by-step, and robust development to accurately determine its functional attributes. Despite these limitations, we have demonstrated that cCR after neoadjuvant therapy was significantly associated with significantly better survival outcomes in MIBC. Pembrolizumab may have a potential role in the neoadjuvant setting as a strategy for bladder preservation, especially in patients unfit for chemotherapy.

## 5. Conclusions

The use of neoadjuvant systemic therapy approaches in MIBC has the potential for bladder preservation without definitive local therapy, particularly in patients achieving cCR. Continued research and advances in strategies incorporating immune checkpoint inhibitors may provide even greater prospects for achieving cCR and successful bladder preservation in the future.

## Figures and Tables

**Table 1:** Demographic characteristics of enrolled patients.

	Pembrolizumab Group	Chemotherapy Group	*p* Value
No. Patient	23	30	
Age (Mean ± SD), years	72.1 ± 12.2	68.6 ± 11.7	0.291
Gender			0.891
Male	18 (78.3%)	23 (76.7%)	
Female	5 (21.7%)	7 (23.3%)	
Stage			0.477
T2N0	13 (56.5%)	14 (46.7%)	
T3N0	10 (43.5%)	16 (53.3%)	
PD-L1 status			0.028
CPS ≥ 10%	17 (73.9%)	12 (40.0%)	
CPS < 10%	5 (21.7%)	10 (33.3%)	
unknown	1 (4.3%)	8 (26.7%)	
Performance status			0.125
0, 1	14 (60.9%)	24 (80%)	
≥2	9 (39.1%)	6 (20%)	
Cisplatin eligibility			0.001
Yes	7 (30.4%)	23 (76.7%)	
No	16 (69.6%)	7 (23.3%)	

SD: standard deviation; PD-L1: program death ligand 1; CPS: combined positive score.

**Table 2:** Adverse effects.

	Pembrolizumab Group		Chemotherapy Group	
No. Patient	23		30	
	Grade 1	Grade 2	Grade 1	Grade 2
Hypothyroidism	2 (8.7%)	1 (4.3%)	1 (3.3%)	0
hyperthyroidism	1 (4.3%)	0	0	0
AST/ALT increase	0	1 (4.3%)	4 (13.3%)	1 (3.3%)
Thrombocytopenia	1 (4.3%)	0	8 (26.7%)	4 (13.3%)
Leukopenia	1 (4.3%)	0	5 (16.7%)	4 (13.3%)
Anemia	2 (8.7%)	0	4 (13.3%)	4 (13.3%)
Diarrhea	1 (4.3%)	0	4 (13.3%)	1 (3.3%)
Nausea	1(4.3%)	0	2 (6.7%)	3 (10.0%)
Pruritis	1 (4.3%)	0	1 (3.3%)	0

Toxicities were graded by Common Terminology Criteria for Adverse Events (CTCAE), version 5.0.

**Table 3:** Recurrence patterns.

	Pembrolizumab Group		Chemotherapy Group	
No. Patient	23		30	
Initial response	cCR (*n* = 12)	Non-cCR (*n* = 11)	cCR (*n* = 11)	Non-cCR (*n* = 19)
NMIBC recurrence	1 (8.3%)	4 (36.3%)	3 (27.3%)	5 (26.3%)
MIBC recurrence	0 (0%)	2 (18.2%)	0 (0%)	2 (10.5%)
Distant metastasis	0 (0%)	1 (4.3%)	1 (9.1%)	6 (31.6%)

NMIBC: non-muscle-invasive bladder cancer, MIBC: muscle-invasive bladder cancer, cCR: clinical complete response.

**Table 4:** Univariate and multivariate analyses for progression-free and overall survival.

	Progression-Free Survival				Overall Survival			
Parameters	Univariate		Multivariate		Univariate		Multivariate	
	HR (95% CI)	*p*	HR (95% CI)	*p*	HR (95% CI)	*p*	HR (95% CI)	*p*
Age	1.002 (0.969–1.036)	0.912	0.984 (0.940–1.030)	0.489	1.005 (0.968–1.042)	0.807	0.980 (0.928–1.035)	0.469
Gender	0.760 (0.327–1.768)	0.524	1.037 (0.414–2.598)	0.939	0.671 (0.250–1.798)	0.427	1.121 (0.379–3.321)	0.836
Stage (III vs. II)	2.054 (0.915–4.615)	0.081	0.903 (0.345–2.3366)	0.836	4.014 (1.424–11.312)	0.009	3.129 (0.864–11.330)	0.082
Cisplatin-ineligible	0.993 (0.450–2.191)	0.985	0.468 (0.138–1.583)	0.222	1.378 (0.533–3.587)	0.508	0.883 (0.215–3.626)	0.863
Pem vs. CT	0.474 (0.203–1.107)	0.085	0.427 (0.151–1.206)	0.108	0.328 (0.116–0.925)	0.035	0.335 (0.092–1.219)	0.097
cCR vs. non cCR	0.127 (0.045–0.357)	<0.001	0.121 (0.038–0.387)	<0.001	0.215 (0.077–0.601)	0.003	0.204 (0.062–0.668)	0.009

Abbreviations: Pem: pembrolizumab, CT: chemotherapy, cCR: clinical complete response.

## Data Availability

Data are contained within the article.
